# Recommendations for the Use of ICT in Elderly Populations with Affective Disorders

**DOI:** 10.3389/fnagi.2016.00269

**Published:** 2016-11-08

**Authors:** Auriane Gros, David Bensamoun, Valeria Manera, Roxane Fabre, Anne-Marie Zacconi-Cauvin, Susanne Thummler, Michel Benoit, Philippe Robert, Renaud David

**Affiliations:** ^1^Département de Neurologie, Centre Mémoire de Ressources et de Recherche, Centre Hospitalier Universitaire de DijonDijon, France; ^2^CoBTek (Cognition—Behaviour—Technology), University of Nice Sophia AntipolisNice, France; ^3^Centre Edmond et Lily Safra pour la Recherche sur la Maladie d’Alzheimer, Centre Mémoire de Ressources et de Recherche, Institut Claude Pompidou, Centre Hospitalier Universitaire de NiceNice, France; ^4^Département de Psychiatrie, Hôpital Pasteur, Centre Hospitalier Universitaire de NiceNice, France; ^5^Département de Santé Publique, Hôpital L’Archet, Centre Hospitalier Universitaire de NiceNice, France

**Keywords:** information and communication technology, serious games, elderly, affective disorders, anxiety, depression, dementia, Alzheimer’s disease

## Abstract

**Objective**: Affective disorders are frequently encountered among elderly populations, and the use of information and communication technologies (ICT) could provide an added value for their recognition and assessment in addition to current clinical methods. The diversity and lack of consensus in the emerging field of ICTs is however a strong limitation for their global use in daily practice. The aim of the present article is to provide recommendations for the use of ICTs for the assessment and management of affective disorders among elderly populations with or without dementia.

**Methods**: A Delphi panel was organized to gather recommendations from experts in the domain. A set of initial general questions for the use of ICT in affective disorders was used to guide the discussion of the expert panel and to analyze the Strengths, Weaknesses, Opportunities, and Threats (SWOT) of employing ICT in elderly populations with affective disorders. Based on the results collected from this first round, a web survey was sent to local general practitioners (GPs) and to all interns in psychiatry in France.

**Results**: The results of the first round revealed that ICT may offer very useful tools for practitioners involved in the diagnosis and management of affective disorders. However, the results of the web survey showed the interest to explain better to current and upcoming practitioners the utility of ICT especially for people living with dementia.

## Introduction

### ICT in Aging Populations

Information and communication technologies (ICT) currently represent pervasive assistive tools for our daily life, and their use is fast growing also in the health domain, where they can improve diagnosis and stimulation strategies in many medical fields. In neuropsychiatry, the development of ICT is changing the use of conventional assessment methods, by progressively integrating computer technologies in their methods of administration, in order to obtain a more reliable and reproducible assessment (Epstein and Klinkenberg, 2001). Currently investigated ICT solutions are mainly numeric solutions (computerized tests, virtual reality (VR) environments, Serious Games) or technology solutions such as wearable sensors that are able to measure physiological parameters (heartbeat, sudation, respiratory frequency, electroencephalogram (EEG), sleep quality, posture and walking quality), or environmental sensors.

For example ICT includes:

– Computerized cognitive tests: they are more and more suited to tactile tablet format and can be used in nursing homes and also at home (Brown et al., 2016).
– Serious games: they allow the management and training of different cognitive deficits (Robert et al., 2016).
– Virtual reality: it is used particularly in the training of visuospatial abilities, memory functions, attention, speed monitoring (Legault et al., 2013) and also in the management of behavioral disorders. Note that adjustments should be taken into account in the VR for the elderly (Robert et al., 2015).
– Wearable sensors and environmental sensors: they can be used for the evaluation of walking abilities (Moufawad El Achkar et al., 2016), to assess the risk of fall (Howcroft et al., 2016), to detect and manage sleep disorders (Lazarou et al., 2016). Wearable sensors can also be used in the assessment and management of affective disorders. For example it has been demonstrated that feedback measurement of the heart rate (HR) by the patient could reduce his stress state (Schaefer et al., 2014). In elderly populations, the use of ICTs may have some drawbacks. First, ICT require IT equipment and maintenance. Second, ICT may be unsuitable for elderly people with physical or sensory disabilities. Third, it has been claimed that there is a major generational limitation considering the potential lack of interest, habits and motivation for new and numeric technologies for elderly people (Naus et al., 2009). However, evidence collected so far did not reveal any particular interest or motivational problem in comparison with young people (Canini et al., 2014). Rather, it has been shown that computerized tests were perceived as more reliable and more comfortable than traditional tests also for elderly people (Kleinman et al., 2001; Greenwood et al., 2006; Millsopp et al., 2006).

Despite several aforementioned limitations, scientific literature on ICT is reporting an increasing number of studies using ICT dedicated to different health-related conditions. In the field of aging, recommendations for the use of ICT and Serious Games have been recently proposed in order to provide new insights and marks in this emerging field for the diagnosis and management of frailty and dementia (Robert et al., 2013, 2014; König et al., 2015).

ICTs may also constitute an added value for the assessment and follow-up of several clinical parameters for which evaluation biases are commonly reported. This is particularly true for neuropsychiatric symptoms, for which there is: (1) a lack of objectivity of current assessment questionnaires and interviews; (2) subjectivity linked to the patient’s self-reports; (3) subjectivity of the rater; (4) cultural differences in symptoms expressivity; (5) agnosia due to cognitive impairment (Orfei et al., 2010); and (6) lack of awareness of disorders due to neuropsychiatric symptoms (Dias et al., 2008). Elderly individuals might additionally present cognitive disturbances like skewing their insight. In terms of cognitive symptoms, a more accurate assessment also appears necessary because it has been shown that patients with complaints not confirmed by tests currently available were at higher risk of developing dementia (Jessen, 2014; Mitchell et al., 2014; Piras et al., 2016).

For affective disorders, several authors have already reported promising results to improve the quality of assessment methods with the integration of physiological parameters of emotional states: voice analysis showing specific patterns in depressed individuals (Mundt et al., 2007; Moore et al., 2008; Scherer et al., 2013), reduced P300-EEG wave amplitude in depressed subjects (Patrick et al., 2006) and P50-EEG wave suppression deficits in patients with bipolar disorder with psychotic symptoms (Schulze et al., 2007), indirect measurement of apathy levels using ambulatory actigraphy (David et al., 2012), increased skin conductance associated to anxiety levels (Marko, 2016), HR decrease associated to major depressive disorders (MDDs; Kemp et al., 2010 and Kemp, 2012).

Despite previous results, affective disorders may have specific characteristics in aging populations that need to be taken into account to provide dedicated ICT-based solutions.

### Clinical Characteristics of Affective Disorders in Aging

In elderly populations, affective disorders may have specific clinical symptoms compared to younger adults (Valiengo et al., 2016) and often constitute early clinical symptoms of dementia that may precede cognitive disturbances. The two most prevalent affective disorders in elderly populations are MDDs and apathy, followed by generalized anxiety disorder. It has also been shown that individuals with amnestic-Mild Cognitive Impairment (MCI) and apathy will more likely progress to Alzheimer Disease (AD; Palmer et al., 2010) and that apathy is a highly reliable predictor for mortality (Spalletta et al., 2015). So, in aging populations, one in four currently have a mental disorder and the most prevalent disorders were anxiety disorders (Andreas et al., 2016). In aging populations, anxiety can often present with atypical symptoms (compared to younger adults) such as yelling, repetitive questioning, wandering, tremor and repetitive movements fear to be left alone, person’s following.

Depression is frequently over diagnosed in elderly populations due to the clinical misdiagnosis with apathy. Apathy is a core symptom of depression, associated to sadness, and is frequently observed in aging. Persons with persistent apathy were less likely to recover from depression than those who remitted from apathy and persistent apathy was associated with worse outcome of depression (Groeneweg-Koolhoven et al., 2016). Apathy is overlapping with other psychiatric and neurodegenerative conditions and it was conceptually ill defined (Starkstein and Leentjens, 2008). A unified definition as well as diagnostic criteria have been proposed directly derived from Marin and Starkstein. The major point is the presence of three distinct dimensions of apathy: the cognitive dimension, the behavioral dimension and the emotional dimension. The presence of apathy may be explained by both age-related brain changes and successive life events (reduced life expectations, retirement, physical frailty) and is often the clinical prevailing characteristic of depression among elderly individuals, whereas mood sadness is, on the contrary, frequently observed among younger adults with depression.

Several age-related biological changes that usually interfere with stress and affective phenomenology, such as the hypothalamo-pituitary axis (likely to be less downregulated in aging) with changes in chronic cortisol secretions, may also partially explain the variability in affective expressivity across ages (Lenze and Wetherell, 2011).

### Towards Recommendations for the Use of ICTs in Elderly Populations’ Affective Disorders

The presence of cognitive disturbances and eventually anosognosia often leads to assessment issues when interviewing elderly individuals with affective symptoms. Current assessment methods for affective disorders, mainly relying on interviews and questionnaires with the patient and the family caregivers, may lack objectivity especially when patient’s and/or caregiver’s insight is impaired, despite this they have usually been validated and are currently used in daily clinical practice. Also higher levels of burden related to care were associated with higher levels of perceived stress and cognitive difficulties experienced which shows that stress and burden of caregivers makes judgment difficult (Luchesi et al., 2016). Additionally, the patient’s presentation during the consultation with the physician may differ from the daily expressed symptoms leading to symptoms misidentification for the clinician. Several symptoms may also have nighttime expressivity that is not easy to assess in daily routine with outpatients. Currently emotions are mainly evaluated using self-questionnaires in which the patient assesses his/her own emotional state.

However, “pencil-paper” tests can encompass differences in instructions depending on the evaluators, consecutive errors in manual data entry, and errors in the transfer of such data to statistical software. To reduce these biases and to obtain a more reliable assessment of a given construct, ICTs have enabled the development of computer-based testing (Epstein and Klinkenberg, 2001). It is recognized that these computerized tests meet certain requirements such as homogenization in terms of similarities in the instructions (Barak, 1999), a playful interface inducing more positive reactions than traditional procurements (Steiner and Gilliland, 2001; Anderson, 2003; Huang, 2006; Naus et al., 2009), reduced errors in data entry (Chuah et al., 2006). However, disadvantages are always highlighted, as the lack of control of the understanding of instructions (Reips, 2000), the lack of accessibility in the case of some sensory impairment, for certain populations or certain cultures (Naus et al., 2009) and the risk of computer-related anxiety (Schulenberg and Yutrzenka, 2004). As for traditional tests (paper/pencil), the international and national commissions (The International Test Commission, 2006) have developed best practice guidelines for the use of computerized tests. They focus both on the equipment used and the methods of administration. Also the computerized tests are encouraged by international commissions regulating the use of tests as they do not alter the relevance and accuracy of the resulting measure.

Other measurements from the ICT and assessing the physiological component of emotions grow more and more, as they avoid the drawbacks and the subjective nature of self-report questionnaires. These measures include skin conductance response (SCR) and HR using wearable sensors placed on the skin. It has been shown a deceleration of HR during negative emotions (Palomba et al., 2000; Bradley et al., 2001) and some studies have reported reduced HR in patients with MDD (Kemp et al., 2010; Kemp, 2012). It was also highlighted that fear was the emotion that provoked the most important variations in the SCR and that it was possible to differentiate patterns of SCRs for fear and sadness (Kreibig et al., 2007). Finally, differences in SCR were found following the presentation of emotional stimuli among individuals with bipolar disorder (Greco et al., 2014) and among individuals with schizophrenia (Park and Kim, 2011) compared to control subjects. ICT allows a more objective recognition of emotional disorders than tests often based on self-administered questionnaires. They have many advantages such as providing a more accurate assessment and less prone to errors, a continuous monitoring and an opportunity to offer non-pharmacologic treatments. However, they do not allow, themselves, to make a diagnosis. They are increasingly used in research settings but they remain still rarely used in current clinical practice. Several studies have started to show the importance of using ICT in clinical trials (König et al., 2015; Pillai and Bonner-Jackson, 2015) and for research purposes for which a lack of objective markers have led to potentially conflicting or inconclusive results. The validation of ICT using existing biomarkers as comparative measures is currently under progress. Recommendations by experts are however required in order to understand which pathologies are concerned, at what stages and the associated risks therein. The Innovation Alzheimer association (IA) is organizing each year, as of 2013, a workshop dedicated to ICT in aging and gathering both experts in the clinical field of aging and from technological field. After each workshop, participating experts published recommendations for the use of ICT in aging. The aim of the present article was to provide recommendations for the use of ICT for affective disorders in aging populations.

## Materials and Methods

### Workshop Strategy

The IA workshop 2015, organized by the Cognition-Behavior-Technology (CoBTek) research team of the University of Nice-Sophia Antipolis, took place in Nice on November 12th 2015.

Using a Delphi method, an expert panel involving at least 25 professionals was chosen to respond to the following aim: “to propose recommendations for the use of ICT in elderly populations with affective disorders”. Four analysts (two psychiatrists and psychologists, two ICT engineers) were involved in the selection of the experts, in the development and analysis of a questionnaire built to drive discussions toward recommendations, and in the editing process of the final report. The analysts and the majority of the experts have been involved in the previous recommendations published during IA workshops 2013 and 2014 (Robert et al., 2013, 2014).

Participants were health care professionals (psychiatrists (*n* = 9), psychologists (*n* = 8), speech therapists (*n* = 3), General practitioners (GPs; *n* = 5)), ICT engineers (*n* = 4) and social workers (*n* = 2). The workshop started with a 3-h plenary session, where recent works related to use of ICT for affective disorders among elderly people with or without cognitive disturbances were presented. For this preliminary review, the keywords “Affective disorders”, “anxiety”, “depression”, “apathy”, “emotion”, “dementia”, “aging”, “frailty syndrome”, “Mild Cognitive Impairment”, “Alzheimer’s disease”, “Information and Communication Technologies”, “Assistive technologies” were searched in the Pubmed and Google Scholar databases.

Experts were then divided into four-person subgroups (with at least one psychiatrist and one psychologist in each subgroup), and asked to answer questions related to the use of ICT for affective disorders as well as to perform a Strengths, Weaknesses, Opportunities, Threats (SWOT) analysis on the same topic.

Many of the questions asked during this face-to-face session were derived from the results of our previous workshops (for which population and severity stages? what type of ICT should be preferred? for what purpose? which affective disorders should be more specifically targeted? which frequency of use? where? with whom?).

Based on the workshop results, a web survey was designed and sent to local general practitioners (GP) involved in the diagnosis and management of both affective disorders and Alzheimer’s disease and related disorders in elderly populations, and to all interns in psychiatry in France (in order to capture the overall feeling of current and future physicians regarding the use of ICTs in their daily practice). GPs are not specialists for the diagnosis and management of affective disorders but they often constitute first-line practitioners and their feedback thus appears to be valuable. The internship in psychiatry in France has a 4-year duration. All interns from 1st, 2nd, 3rd and 4th year received a web invitation to participle in the survey. All GPs working in the city of Nice also received the web invitation to participate in the survey. Participants were presented with the list of questions and items employed during the Workshop and asked to rate each item on a 0–2 scale (0 = not adapted at all; 1 = neutral; 2 = very adapted).

One-hundred and forty-one interns in psychiatry and 34 GPs have responded to the survey.

### Population of Interest

The following diagnostic criteria related to specific medical conditions with cognitive decline were considered in the writing process of the present recommendations for the use of ICT: frailty syndrome (Fried et al., 2001), MCI (Petersen, 2004), Alzheimer’s disease (NINCDS-ADRDA; McKhann et al., 1984). From Fried the frailty syndrome is a well-defined syndrome with biological underpinnings and clinical manifestations related to negative energy balance, sarcopenia, and diminished strength and tolerance for exertion. Regarding the definition of Alzheimer’s disease and related disorders, we decided to consider both initial and more clinical criteria and as well as more recent and advanced criteria embedding biomarkers, physicians with strong expertise in this field of dementia and non specialist physicians (interns in psychiatry, GPs). The severity of the cognitive decline was defined, according to the scores on the Mini Mental State Examination (MMSE; Folstein et al., 1975) as follows: mild cognitive decline (21 < MMSE < 26), moderate cognitive decline (16 < MMSE < 20), severe cognitive decline (MMSE < 15). Psychiatric states were defined according to the DSM-V criteria for mood disorders, anxiety-related disorders and Post Traumatic Stress Disorders. Diagnostic criteria for apathy were considered (Robert et al., 2002). Individuals considered for these recommendations were predominantly 65 years. This is the common age proposed to distinguish between early onset dementia and common onset dementia. In order to facilitate the collaborative brainstorming, this cut-off was considered for all diseases despite the fact that age for early onset disturbances may vary according to recommendations proposed for each psychiatric disorder.

## Results

### From The Expert Panel

#### General Questions

In each four-participant subgroup there was at least one psychiatrist and one psychologist. Other participants included physicians (GPs, neurologists, geriatricians), ICT engineers or social workers. Participants were asked to discuss a set of several questions regarding the use of ICT for the diagnosis, follow-up and management of affective disorders in elderly populations, with and without associated cognitive disturbances (from Mild Cognitive Impairment to Dementia). All responses were then synthesized and summarized as follows.

### Which Population or Severity Stages

Subgroups reported an overall interest in using ICTs for individuals with Mild Cognitive Impairment, frailty syndrome (Fried’s criteria), or mild to moderate dementia syndrome (MMSE between 26 and 16), but did not recommend using ICT for severe stages of dementia (MMSE < 15). The severity of the cognitive decline is expected to decrease the ICT acceptability (lack of understanding of ICTs utility, feeling of intrusive devices) and to induce anxiety or delusion. Also, patients with severe cognitive decline may be unable to give an opinion about the device’s functioning, and have usage difficulties, which make the device not appropriate to their needs (Faucounau et al., 2009).

### What Type of ICT

Experts reported that all types of devices and/or solutions could be considered, but should take into account the subjects’ sensory impairments, cultural aspects and associated cognitive disturbances.

For example, subjects with hearing impairment should not be assessed using speech analysis solutions or ICTs with verbal instructions. Subjects with hallucinations or delusions should not be evaluated using skin sensors or environmental sensors.

Results from recent studies have shown that several stimulation-oriented ICT could likely improve affective affects, such as using serious games on tablet showing interest for apathetic individuals (Manera et al., 2015) or exergame showing increased positive emotions and interest (Ben-Sadoun et al., 2016). In various medical situations, the use of commercial video games for rehabilitation showed similar results compared to conventional rehabilitation methods but increased patients’ motivation (Bonnechère et al., 2016). More generally, the gamification of cognitive assessment tools and cognitive trainings is likely an interesting way to increase patients’ motivation and engagement (Lumsden et al., 2016).

Regarding affective disorders more specifically, VR solutions have shown interesting results for the management of anxiety-related disorders in adults (Parsons and Rizzo, 2008; Bruce and Regenbrecht, 2009; Yeh et al., 2009; Taffou et al., 2012). VR-based serious games could also provide benefits in reducing depression levels among elderly individuals (Burdea et al., 2015). The overall literature on ICT for elderly population remains however currently scarce.

### What Purpose (Diagnosis, Follow-Up, Management)

ICT-based solutions could be used for the assessment in elderly populations with affective disorders through more objective assessments, including devices or solutions which are able to capture physiological parameters related to emotional states.

ICTs could be used for the assessment of individuals with limited mobility or living far from specialized centers.

ICTs could be used for in-home monitoring in order to have continuous measures (24/7-monitoring), as well as for the regular follow-up of outpatients consulting in specialized settings.

ICT could be used for the non-pharmacological management of affective disorders.

For example, VR has been used extensively in the treatment of phobias such as claustrophobia (Bruce and Regenbrecht, 2009), the phobia of animals (Taffou et al., 2012) or during post-traumatic stress disorder (Rothbaum et al., 2010).

### For which Affective Disorders

Experts reported that anxiety disorders should be the main target of ICT use, but also depressive symptoms and apathy may represent interesting targets.

However experts believe that psychotic disorders are not prime targets for ICT, as well as disorders with significant cognitive deficits.

### Which Frequency

Experts reported that ICT could be used on demand for individuals at home, and likely with a more organized schedule for individuals in hospitals or long-term care facilities due to staff constraints.

The experts suggested that in some cases the frequency of use should be indicated by the doctor or be defined in the context of a medical prescription.

However, home video sensors for monitoring must be installed for sufficient time to be able to identify regular events.

### Where

Participants proposed several locations for the use of ICT: at home, in hospital, in long-term care facilities, as well as waiting room of GP’s office.

The choice of ICTs will depend on the location:

– At home, environmental sensors can be recommended to monitor several behavioral changes (nighttime behaviors, wandering) that could indirectly reflect emotional changes.
– At the hospital, wearable sensors are the most recommended in order to provide objective and controlled assessments.
– In GP’s offices, computerized tests are more recommended because of the possibility to control the understanding of instructions.

### With Whom

Several experts reported the need to use ICT after medical prescription to give a frame in the use of these new types of devices. The importance of the GP has been highlighted in the use of ICT. Several reasons were advanced: (1) GPs are first-line physicians and could contribute to improve prevention and early diagnosis of affective disorders using objective methods involving ICTs, despite their lack of training in the screening of affective disturbances; and (2) GPs’ consultations are usually too short to fully capture patients’ global medical status. ICT could be used, for example, in the waiting room, to start recording several relevant medical parameters that could be provided to the GP during the consultation. Also, involving GPs, or caregivers, will allow them to explain to the patients how they can use ICTs, at what times and remind them the instructions required. This would enable to customize ICT to each patient. In care facilities, use of ICT should be done under staff supervision.

### SWOT Analysis

We also asked the panel to conduct a SWOT analysis for the use of ICT in affective disorders among elderly individuals. A summary of the SWOT analysis is presented in Table 1.

**Table 1:** **Strengths, Weaknesses, Opportunities, and Threats (SWOT)**.

Strenghts	Weaknesses
Objective evaluation	Devices too sophisticated and complicated to use
Interface adapted and tailored to the user,	Expensive equipments
Possibility to use at home	Addiction.
Real-time feedback delivery for the user and the professionals	Risk of overdiagnosis
Improve screening and early diagnosis, improvement of mass screening	Risk to induce new symptoms for advanced dementia stage (anxiety, delirium, persecution)
Possibility to provide prolonged or continuous evaluation	Risk of decreasing social, familial and outdoors activities

**Opportunities**	**Threats**

Geographic equity	Ethical challenges
Adapted to the new generations	Perception that ICT will replace clinicians, disappearance of human relations
Provide homogeneous therapeutic actions in care structures, provide therapeutic actions on patients’ living place	Negative representation (intrusive devices and privation violation)
Possibility to provide professional training	Risk of standardization, separate affective disorders from temper, personal history, traumatic life events
Embedded physiological measure of affective symptoms (respiratory and heartbeat, sudation, …)	Lack of consensus on specific markers for affective disorders

#### Strengths

##### Objective Evaluation

It is recognized that ICT allows a better homogenization in the awarding and issuing instructions during evaluations (Barak, 1999). It also limits the raters’ differences.

Finally, ICT allow more objective evaluations because they limit the consecutive errors deriving from a manual data entry, and allow an easier transfer of data to statistical software (Chuah et al., 2006).

##### Interface Adapted to the User

In the context of ICT it is possible to create interfaces adapted to the users. One of the advantages of ICT is that it is possible to individualize and personalize the evaluation. Adaptation is important for elderly people because they can have sensorial problems (auditory, visual) and memory problems, which require repeating instructional cues.

##### Possibility to Use at Home

The home-based and independent practice would contribute to reduce the burden to public healthcare system. ICT can promote activity automation, and generalization of the learned skills to every-day behavior. The possibility to carry out an autonomous activity can also lead to mood improvements and self-esteem enhancement. Finally, the possibilities to use ICT at home represent an opportunity for a safe testing and training environment, minimizing risks due to travel.

##### Real-Time Feedback Delivery for the User and the Professionals

ICT offer the therapists and caregivers the possibility to record and visualize the activity immediately, while the classical evaluation of patient performances typically involves a *post hoc* examination. The advantage is that ICT allow the patient to see directly his/her mistakes, and correct them. So, therapists can give the results of their assessment immediately, and give directions to the patient immediately after the conclusion of the tests.

##### Possibility to Provide Prolonged or Continuous Evaluation

The frequency and duration of medical consultations are limited, which may bias the assessment results because the patient, for instance, may be tired during the consultation. Motivation is important not only for a successful care but also for the scores obtained during an evaluation. A continuous and repeated analysis can avoid the bias due to the moment when the evaluation took place.

##### Improve Screening and Early Diagnosis, Improvement of Mass Screening

The ICTs for the larger dissemination of evaluations may allow evaluating a larger portion of the population compared to conventional ratings, which require the presence of a therapist. Similarly, ICTs, by finer assessments, are more sensitive methods.

#### Weaknesses

##### Devices too Sophisticated and Complicated to Use

Many elderly people are not used to interacting with high-tech interfaces, and the initial approach with ICT may cause them a high cognitive load. This high cognitive load can represent a bias for the assessment because it can make the task more difficult than what it actually is. Also to employ ICT solutions it is often necessary to connect cables and modify setups, which may be too difficult for non-expert users, especially for the elderly. Finally it may be also difficult for the therapists to use the applications to visualize performance results and analyze data.

##### Expensive Equipment

Equipment for ICT is a stuff that we already have at home such as tablets, television, PC and laptops. However, some ICT require the purchase of less common and more expensive materials such as CAVE or immersive screen. Equipment costs may risk limiting the use of ICT to people who have the most money, thus creating discrimination.

##### Negative Representation (Intrusive Devices and Privacy Violation)

Some ICT, especially for assessment, require the installation of video-cameras or sensors in patients’ homes. These sensors can be perceived as intrusive in the patient’s privacy, in particular when they must be installed in the bedroom to evaluate sleep disorders.

##### Addiction

As with video games, new technologies can create addictions for patients, especially if their use is not governed by rules and indeed, currently, the use of new technologies in patients’ homes cannot be controlled, which makes it dangerous.

##### Risk of Overdiagnosis

It appears that the use of ultra-sensitive diagnostic techniques is most often accompanied by a significant increase in positive diagnosis, seeming to be more of overdiagnosis. ICT, by finer assessments could thus result in increased diagnosis of affective disorders and therefore an overdiagnosis.

##### Technophobia, Risk to Induce New Symptoms for Advanced Dementia Stage (Anxiety, Delirium, Persecution)

Several types of ICTs can lead to other symptoms in a patient. For example, the installation of cameras at a patient’s home can cause delusions of persecution, the use of VR can lead to a phobia or anxiety, use of sensors on the skin can generate hypochondria. ICTs are intrusive sensors and are therefore particularly at risk to generate a secondary pathology.

##### Risk of Decreasing Social, Familial and Outdoors Activities

The use of ICT is often individual and therefore can generate a feeling of not needing the others, thereby reducing external activities and social relations in the patient. Also, very few ICT are used outdoors and therefore it does not encourage the patient to go outside.

#### Opportunities

##### Geographic Equity

Patients who live far from the cities go less often to the doctor or the therapist and therefore have less regular care than people living in the city. ICTs can reduce this inequality by enabling patients living far from the cities to access care without having to move and therefore to be able to have an evaluation and a more regular care.

##### Adapted to the New Generations

The elderly in the future will be the youth of today who are more accustomed to new technologies than to the “paper and pencil” tasks. Indeed the new generations are faster with ICT. New technologies are intuitive to them and are therefore better for the assessment and the training.

##### Provide Homogeneous Therapeutic Actions in Care Structures; Provide Therapeutic Actions on Patients’ Living Place

It is difficult to assess and take care of a patient in an environment different from where he/she lives. Indeed, the patient can succeed in the classical “paper and pencil” assessments, but no have autonomy in real activities of daily living, or be autonomous in daily life and still fail in “paper and pencil” assessments. ICT, analyzing the behavior at home, can allow a more accurate assessment of patient skills. Furthermore, the use of ICT in nursing homes can enable taking care of the patients in an automated and therefore more regular manner, overcoming the problem of the limited care giver availability.

##### Possibility to Provide Professional Training

ICTs, with wide distribution, can afford to train a large number of professionals at the same time, where they want and when they want. Thus, these technologies make easier access to assessment methods and specific support that were previously reserved for specialists.

##### Embedded Physiological Measure of Affective Symptoms (Respiratory and Heart beat, Sudation, …)

Physiological measurements allow an objective assessment of affective disorders and are not dependent on factors such as social status or culture. In addition, these signals, such as the heartbeat, or the skin conductance, allow for a more accurate and sensitive measurement.

#### Threats

##### Ethical Challenges

The use of ICT poses a number of ethical questions such as:

– The respect of privacy and private life, the processing of the images (using video sensors).
– The data processing and storage.

These ethical questions are even more compelling for older and frail people who cannot always accurately understand the functioning of ICT.

##### Perception that ICT will Replace Clinicians, Disappearance of Human Relations

Some uses of ICT can promote short and little personalized exchanges between patient and therapist. In those situations where therapists have little time for more informal exchanges, the patient can develop a sense of isolation, an erosion of the sense of caring and more overall a formalization of exchanges.

##### Risk of Standardization, Separate Affective Disorders from Temper, Personal History, Traumatic Life Events

For economic reasons and/or for easier and faster usability, ICTs might be used in a standardized way that could minimize the interest of ICTs to propose tailored assessments and managements.

##### Lack of Consensus on Specific Markers for Affective Disorders

Several ICT use the video sensors to evaluate affective disorders. These sensors analyze behavioral disorders but the specific behaviors reflecting emotional problems still require a better specification and precision. Similarly, data on voice markers, are so far not numerous enough to be certain of their reliability.

### From The Web Survey

One-hundred and forty-one interns in psychiatry and 34 GPs responded to the survey.

The questionnaire results showed that, in the general population, the pathologies considered the most difficult to identify for interns in psychiatry were bipolar disorder (28.6%), followed by emotional lability (25%) and generalized anxiety disorder (14.3%). GPs identified Obsessive Compulsive Disorder (38.2%), phobic disorders (38.2%) and depressive episode (23.5%) as difficult diagnoses.

Around 25% of participants (both in interns and GPs subgroups) found ICTs relevant for the detection of affective disorders (with higher rates of responses considering ICT inappropriate for affective disorders). Among positive responders, the use of computerized tools was considered more relevant compared to wearable sensors. ICTs were considered as promising tools for the patients’ follow-up, after the diagnosis has been made, as well as for non-pharmacologic strategies (see Table 2).

**Table 2:** **Use of information and communication technologies (ICT) in the general population**.

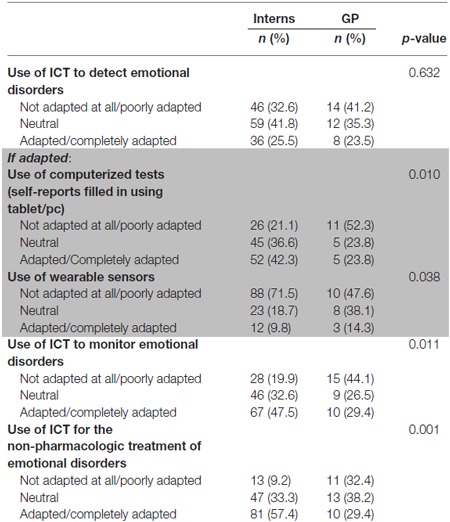

Considering elderly populations and subjects living with dementia (Table 2), interest of using ICT, for both interns and GPs, was below 30%. For the majority of GPs (23.8%) and interns (42.3%), the use of computerized tests was more adapted than using wearable sensors. Interns seemed significantly more likely to use computerized tests than GPs (*p* = 0.010). The majority of interns found adapted or very adapted to the use of ICT in the monitoring of emotional disorders (47.5%), unlike GPs who were found to be poorly adapted (44.1%; *p* = 0.011).

The difference of opinion of interns and GPs was also significant in the relevance of using ICTs as treatment (*p* = 0.01). The majority of interns (57.4%) considered this use as adapted to very adapted, while 32.4% of GPs were found to be poorly adapted.

Among the most difficult disorders to identify, GPs found that ICTs are irrelevant, whether for obsessive-compulsive disorder (54.5%), phobic disorders (44.1%) or depressive episodes (63.6%).

We noted a significant difference with interns who found that the use of ICT can help in screening depressive episodes (*p* = 0.048), phobic disorders (*p* < 0.001) and also obsessive compulsive disorders (*p* < 0.001). However, according to interns, the use of ICT is not relevant for assessing emotional lability (40.7%), bipolar disorder (38.6%) or generalized anxiety disorders (43.8%).

**Figure 1:**
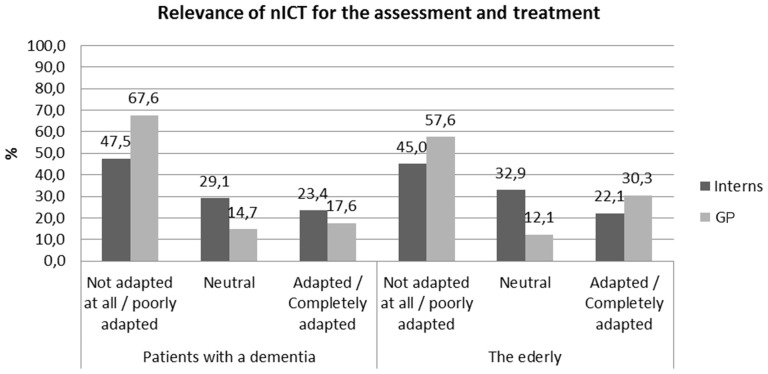
**Relevance of information and communication technologies (ICT) for different affective/emotional disorders**.

Finally, the survey revealed that interns and GPs find the use of new technologies more adapted for healthy elderly people than for AD patients (Figure 1).

### Recommendations

Several major conclusions can be drawn after the general questions, SWOT analysis and web survey.

First, ICT could contribute to provide better recognition and earlier diagnosis of affective disorders in elderly populations, because ICT could be designed to specifically recognize affective symptoms using specific and dedicated algorithms. However, in order to become clinically accepted, therapists should undergo ICT trainings to learn how to employ them correctly and in a standardized way.

Second, first-line physicians, mainly GPs, could benefit from ICTs: (1) as they are usually “reference physician” (and officially declared to the health regulatory authorities for health coverage), receive more patients compared to specialists, and often consult in patients’ home; and (2) as they usually lack specific training to recognize affective symptoms despite being first-line practitioners. In this line, use of ICT aimed at recognizing affective symptoms could help GPs in their daily practice but could also contribute to large population screening. The limited amount of responses gathered from GPs as well as several comments noticed after our web survey tend to indicate an overall lack of knowledge on ICT capabilities for health management. In this line, increased diffusion of ICT is required among physicians.

Third, ICTs for affective disorders should embed measures of physiological parameters linked to affects and emotions such as respiratory frequency, heartbeat, skin conductance, and so on. However, more specific markers of affective disorders are likely needed to be recognized to better participate in the assessment and follow-up process of affective symptoms in addition to current clinical markers. Participants reported the use of ICT at home as a strength for affective disorders. Developers and clinicians should thus also discuss the use of devices with ambulatory affective markers that could be sensitive to at-home changes. Participants in the web survey did not consider the use of wearable sensors as adapted, especially among interns in psychiatry.

Fourth, ICTs could be helpful in prevention, diagnosis and non-pharmacologic treatment for several affective symptoms (mainly anxiety disorders and depressive symptoms). Experts pointed out that ICTs could potentially have a negative impact for individuals with advanced dementia stages (increased risk of delirium avec persecution, increased consecutive anxiety) whereas they appear to be adapted for preclinical, frail and early to moderate stage demented individuals.

## Discussion and Future Research Directions

We found important differences between the reports of the expert panel and GPs and interns interviewed in the web survey. Indeed, the experts recommended the use of ICT for patients with AD, while the majority of GPs and interns suggested that ICTs are not adapted for AD patients.

Similarly, while experts recommend the use of ICTs for evaluation, monitoring and treatment, interns found ICTs adapted only for the monitoring and treatment and GPs, more generally, found ICT irrelevant.

Nevertheless the percentage of neutral responses showed the interest of better informed interns and GPs on ICT interest and opportunities. Indeed we can assume that the persons interviewed found use of ICT not adapted to individuals with AD because, unlike the experts, they do not use ICT’s in their daily practice and they are poorly aware of the available tools.

Opinions are divergent between GPs and interns particularly for the use of computerized tests for diagnosis. We can assume that the interns are part of a generation accustomed to these type of tests, whose use has grown significantly in the clinical practice in recent years.

Using wearable sensors is particularly not adapted to AD patients according to interns (71%). This skepticism may derive from the fact that interns, most likely, use ICT very rarely in their daily practice. But this skepticism may derive also from the fact that wearable sensors can be particularly difficult to use with patients often agitated, presenting aggressive behaviors and which have aberrant wondering behaviors.

In the web survey it is interesting to note that disorders were considered the most difficult to identify by GPs and were those most easily identified by interns, with a significant difference for depressive episode (*p* < 0.001), phobic disorder (*p* = 0.003) and obsessive compulsive disorder (*p* = 0.004). This may be due to the fact that, being complex pathologies, they are well studied in Psychiatry.

Finally, several topics could be discussed on:

–Psychotropic medications:

Several psychotropic medications have shown a deleterious impact on cognitive abilities (e.g., long term exposition to benzodiazepines (Shash et al., 2016), antipsychotics and many agents with anticholinergic effect) as well as on emotional and affective states (e.g., negative effect of SSRIs and antipsychotics on motivation often leading to increased levels of apathy (Padala et al., 2012; Dell’Osso et al., 2016 ); psychic retardation with antipsychotic and benzodiazepines; effect of corticoids and cancer agents on mood and vigilance).

Thus, all types of psychotropic medications known to have negative impact on vigilance and motivation could potentially reduce the engagement of patients in using ICTs when proposed as long-term monitoring tools and/or non-pharmacologic approaches (i.e., stimulation with serious games).

Additionally, ICT represents an opportunity to provide non-pharmacologic approaches (through serious games for example) in neurodegenerative disorders and affective disturbances (Bruce and Regenbrecht, 2009; Taffou et al., 2012) or during post-traumatic stress disorder (Rothbaum et al., 2010) and could help reduce the use of psychotropic medications.

–Role of caregivers:

Experts have discussed the role of professional and family caregivers when using ICT. Several aspects could be discussed: what is the influence of caregivers on the willingness of patients to use ICT? To our knowledge, this aspect lacks scientific references due to the recent emergence of ICT. Based on our experience, the use of ICT in long-term care facilities often requires the prompt of the professional caregivers, especially for people with severe dementia. For community-dwelling individuals, the feeling is similar and the use of ICT is often influenced by the presence of a cognitively healthy family caregiver living with the patient. Regarding affective disturbances more specifically, many emotional disturbances (anxiety-related disturbances, post-traumatic stress disorder) are often comorbid with depressive episodes and associated to social isolation, lack of interest and lack of motivation. Lack of motivation is associated with lack of initiation in activities that would therefore reduce patients’ engagement in stimulating activities (such as using serious games, for example, that have to be considered as part of ICT). Thus, the presence of affective disturbances could likely decrease the patients’ willingness to participate in stimulating activities. ICT could potentially offer a way to engage in socially stimulating activities (Coathup et al., 2016).

–Impact of setting:

The point has been discussed by the experts but the literature on ICT is also lacking. According to our experience, the presence of professional caregivers (when trained) in long-term care facilities might increase the patients’ stimulation for activities and potentially the use of ICT designed for stimulation (such as serious games). Additionally, many assistive ICT designed to improve patients’ full-time monitoring in long-term care facilities should offer more availability for professional caregivers by decreasing the time devoted to patients’ surveillance, thus potentially increasing interactions between patients and caregivers for stimulating activities.

## Author Contributions

AG: drafting the work, conception, interpretation of data. DB: drafting the work, conception, acquisition of data. VM: revising the work, design, interpretation of data. RF: drafting the work, analysis of data. A-MZ-C, ST and MB: drafting the work, acquisition of data. PR: revising the work, acquisition of data. RD: drafting and revising the work, design, interpretation of data.

## Conflict of Interest Statement

The authors declare that the research was conducted in the absence of any commercial or financial relationships that could be construed as a potential conflict of interest.
